# What is wrong with reductionism? On the normative nature of mental disorder

**DOI:** 10.3389/fpsyg.2014.00122

**Published:** 2014-02-14

**Authors:** Markus Rüther

**Affiliations:** ^1^Forschungszentrum Jülich, Institut für Neurowissenschaft und MedizinGermany; ^2^Max-Planck-Gesellschaft Münster, Institut für Ethik, Geschichte und Theorie der MedizinGermany

**Keywords:** concept of mental disorder, anti-reductionism, irreducibility, values, normativity

The subject of Marco Stier's article seems to be well-known, as he addresses a prominent topic in the philosophy of psychiatry: the normative nature of mental disorders. Of course, Stier does not attempt to cover this issue extensively, since he focuses rather on the Irreducibilty-Thesis (IT) and tries to show that “psychiatric diseases are irreducible to the brain even if the mental as such may in principle be reducible” (p. 2). Admittedly, such anti-reductionism is not an uncontroversial position (for an overview of the debate see Perring, 2010, ch. 3). Here is not the place to go into too much detail in dealing with this approach, even though I have expressed my sympathies elsewhere (Rüther, in press). Much rather, I want to pick out three points which, in my opinion, wrongly find little or no consideration. The first point concerns a position which Stier ties to IT, namely social constructivism; the second point concerns the argumentative strategy used in defending IT. The third point finally is a general comment on the orientation of the debate, i.e., the question of what we should discuss when talking about the normativity of mental disorders.

## The constructivist-thesis

It is a unique feature of the text that Stier does not stick only to the far-reaching topic of normativity, but also makes a connection to debates on objectivity. He concludes that if IT is true, “psychiatric disorders are not out there and not [to] be understood as objectively discoverable entities that can always be separated from each other” (p. 8). But what are they then? According to Stier, we are dealing with a social construction. For, “[N]o inner feeling has a sticker on it that reads “I'm a disorder!” We have to write those stickers ourselves and attach them to certain feelings and behaviors” (p. 2, also p. 3, p. 7). If we stick to these ideas, then it follows that we have to understand the concept of mental illness as non-objective or, put positively, as a social invention. This last thesis we may call the claim of the social construction of mental illness (CT).

At this point a number of follow-up questions arise, for instance, how one is to spell out the construction metaphor and how plausible this “spelling out” actually is. How does Stier deal with these matters? At times, it sounds as though Stier justifies CT by invoking a conceptual relation to IT: “Again, if the boundary between normality and mental disorder is a social construction […], then the, disorderness' of a condition cannot be found on - and hence not be reduced to - the neuronal level.” (p. 4) But it is clear (and hardly worth mentioning) that such a relation does not exist. In recent years, philosophers have drawn attention to the fact that the notions of normativity and objectivity are different (see the locus classicus Wiggins, 1976; McDowell, 1985, 1987). Thus, it requires separate arguments to defend CT. In Stier, too, we find passages in which this is acknowledged and at least one such argument is to be found. This argument is well-known in the debate and starts with the descriptive assumption that the question what mental illness happens to be, is relative to a given context. This can be seen in various discourses, for instance, by researching the causes (p. 6), diagnosis and explanation (p. 6 et seq.), or the experiential quality of mental ilness (p. 7 et seq.). However, we should recognize the following state of affairs: It seems suspect to make claims about the nature of mental illness by referring merely to of how mental illness is understood de facto. Empirical judgments and judgments about the nature of mental illness are independent of one another. A difference that Stier explicitly concedes and makes use of himself (see his defense of IT below). So, perhaps we should understand the pointer toward empirical variance in another way, maybe not as a direct inference to CT, but as a call for explanation. In this case, one might claim by abduction that CT is the *best* explanation for the factual diversity. Indeed, we can find several indications for such a reading, for instance, when Stier explains the “extreme variance of prevalence rates for, e. g., social anxiety disorder” (p. 7) by the fact that psychiatrists themselves define what an illness is by “put[ing] up a sign that reads, Attention, you are leaving the normal sector!'” (ibid.) But is such an inference really persuasive? I have doubts, in particular, because I cannot see that the objectivist counter-position has a *worse* explanation (see e.g., Rüther, 2013, ch. 13.1). Why shouldn't we claim that many divergences are based on distorted patterns of perception, for instance, on psychological, semantic or logical fallacies? In most cases this would be even more intuitively cogent than using the metaphor of construction. In this manner, it seems that the constructivist can at most achieve an argumentative draw. Ideally, he can offer an explanation that is comparable in quality to that of the objectivist. But if things are like this, doubts arise whether the difference-argument for CT actually can reach its aim. The mere fact that different beliefs about mental illness exist is not a sufficient reason to take constructivism to be true.

## The argument for anti-reductionism

As we have seen, Stier's main aim is to defend IT. He writes: “All I want to show is that mental disorders cannot be determined in a purely physical way” (p. 2). This is not a modest aim, but rather a highly complex one and the literature on the topic is vast. Seen from this angle, one might expect a detailed engagement with proponents and opponents of IT. But taking a closer look at the text, we get a different picture. For the most part, the text points to fields and areas in which we can assume that psychiatry carries heavy normative baggage (key words are: “frame of reference,” “normative dimensions of psychiatry”). Accordingly, the text does not, strictly speaking, argue for IT, but offers a description of the normative phenomena at issue. Of course, this description is also a comprehensive project, and Stier's extensive and sophisticated comments are worth noting in this manner. Yet, pointing to our phenomenology is not sufficient for his claim that IT is true. What we do need is not only a description of the data, a common ground to which reductionists and non-reductions can apply their approaches. We also need *a reason* that counts against reduction. But at times, Stier comes close to the claim that assembling normative preconditions is enough. For he speaks of the “normative bedrocks of mental disease” (pp. 2–8) and claims that “[i]t is in principle impossible to get rid of this normative aspect of the task, even if the underlying biological mechanisms of a particular behavior or experience were completely known” (p. 4). In this regard, Stier owes us a reason why the irreducibility of the normative might be the best explanation for the discussed phenomenon, particularly if one takes into account the present state of the debate and the extensive literature on the various counter strategies of the reductionist.

## The focal point of the debate

However, why should we bother with the complicated dialectics between reductionism and anti-reductionism in psychiatry at all? Might it not for some reason be sufficient to point to the normative preconditions and assume that this is enough? Surely, for some purposes it might be sufficient that we do not have to get entangled in the reductionist counter arguments. Nevertheless, I would suggest that there is at least one reason to deal with these arguments. This is mainly that we can get a grip on the matter of what really is at issue when we spell out the dialectic between the two opponents. And if we do so, we can see that both parties, at bottom, are actually arguing about different conceptions of *how* to analyse philosophical problems. This is, of course, not a novel conception of what the debate on reductionism is about (see e.g., Keil, 2008). The reductionist is, at least in a common reading, a philosopher who tries to accommodate the phenomena in question into a natural framework which is mainly investigated by the natural sciences. In contrast to that, the anti-reductionist is suspicious about this unification and rejects this as a vicious simplification that has unbearable costs, e. g., it leaves something important out or, even stronger, comes close to a self-contradiction. Framed in this way, we can see that the stakes are higher than we might previously have assumed, for now we are concerned not only with questions internal to the philosophy of psychiatry, but with fundamental questions about the nature of philosophy and its methodology. Perhaps, this background also explains Stier's implicit target and the intensity with which he, and others with him, reject reductionism. Of course, questions like these are complicated and call for further explanations of the terms already used, for instance, the reductionist terms “method,” “natural framework” or “modern science.” But in any case, it is worth being aware that these explanations also lead us away from the narrow philosophical context of psychiatry and point toward a metaphilosophical reflection.

## Conclusion

What has been said so far? First, it was shown that Stier's constructivist claim is not sufficiently supported. It can neither be deduced conceptually nor is the proposed auxiliary argument of any help. Here we obviously need further arguments in order to support it.

Second, it was noted that the claim of irreducibility is explicated but not defended. It was shown in which way normativity plays a role, but not why it resists any form of reduction. Here one should draw out the dialectic and hear the reductionist position.

Third, it should have become clear that the debate on reductionism is not only a debate specific to the philosophy of psychiatry, but belongs to the wider field of metaphilosophy. The debate about IT is, at its core, concerned with the nature and methods of philosophy. Thus, if one wants to follow up on the debate about normativity in psychiatry, here might lie a field for promising future research.
